# Distinguishing HapMap Accessions Through Recursive Set Partitioning in Hierarchical Decision Trees

**DOI:** 10.3389/fpls.2021.628421

**Published:** 2021-02-03

**Authors:** Wenchao Zhang, Yun Kang, Xiaofei Cheng, Jiangqi Wen, Hongying Zhang, Ivone Torres-Jerez, Nick Krom, Michael K. Udvardi, Wolf-Rüdiger Scheible, Patrick Xuechun Zhao

**Affiliations:** Noble Research Institute LLC, Ardmore, OK, United States

**Keywords:** genome-wide association study, genotype, HapMap accession, homozygous, hierarchical decision tree, INDEL, set partitioning, SNP

## Abstract

The HapMap (haplotype map) projects have produced valuable genetic resources in life science research communities, allowing researchers to investigate sequence variations and conduct genome-wide association study (GWAS) analyses. A typical HapMap project may require sequencing hundreds, even thousands, of individual lines or accessions within a species. Due to limitations in current sequencing technology, the genotype values for some accessions cannot be clearly called. Additionally, allelic heterozygosity can be very high in some lines, causing genetic and sometimes phenotypic segregation in their descendants. Genetic and phenotypic segregation degrades the original accession’s specificity and makes it difficult to distinguish one accession from another. Therefore, it is vitally important to determine and validate HapMap accessions before one conducts a GWAS analysis. However, to the best of our knowledge, there are no prior methodologies or tools that can readily distinguish or validate multiple accessions in a HapMap population. We devised a bioinformatics approach to distinguish multiple HapMap accessions using only a minimum number of genetic markers. First, we assign each candidate marker with a distinguishing score (DS), which measures its capability in distinguishing accessions. The DS score prioritizes those markers with higher percentages of homozygous genotypes (allele combinations), as they can be stably passed on to offspring. Next, we apply the “set-partitioning” concept to select optimal markers by recursively partitioning accession sets. Subsequently, we build a hierarchical decision tree in which a specific path represents the selected markers and the homogenous genotypes that can be used to distinguish one accession from others in the HapMap population. Based on these algorithms, we developed a web tool named MAD-HiDTree (Multiple Accession Distinguishment-Hierarchical Decision Tree), designed to analyze a user-input genotype matrix and construct a hierarchical decision tree. Using genetic marker data extracted from the *Medicago truncatula* HapMap population, we successfully constructed hierarchical decision trees by which the original 262 *M. truncatula* accessions could be efficiently distinguished. PCR experiments verified our proposed method, confirming that MAD-HiDTree can be used for the identification of a specific accession. MAD-HiDTree was developed in C/C^++^ in Linux. Both the source code and test data are publicly available at https://bioinfo.noble.org/MAD-HiDTree/.

## Introduction

A HapMap project aims to develop a haplotype map of a genome of interest and describe the common patterns of genetic variations among individuals. This always requires sequencing and genotyping hundreds, even thousands, of individual lines/accessions. Investigation of the sequence variation among multiple accessions can facilitate the study of gene function by genetic screening (Page and Grossniklaus, 2002; Østergaard and Yanofsky, 2004), and identification of the sequence variants that affect phenotypic traits by association mapping (Gibbs, 2003). Sequence variation data usually are acquired by genome-wide sequence alignment (Gollery, 2005) and subsequent genotype calling (Nielsen et al., 2011). Due to the limitations of current DNA sequencing technology (Kircher and Kelso, 2010) and imperfect methods for alignment and genotype calling (Nielsen et al., 2011, 2012), the genotype of some genetic variants cannot be clearly called and are recorded as missing or unknown. Additionally, genetic and phenotypic segregation can be observed in their descendants, mainly due to the considerably high heterozygosity (HET) rate. It has been reported that the genome-wide mean HET rate estimated by Single Nucleotide Polymorphism (SNP) database in a well-designed HapMap data may be as high as ∼0.2 (Li et al., 2007; Anderson et al., 2010).

In the past decade, great success has been achieved in the application of population genetics and genome-wide association studies (GWASs) (Visscher et al., 2012, 2017). GWAS has become a powerful approach to identify genotype–phenotype (G2P) associations (Tam et al., 2019) with many tools developed (Yang et al., 2011; Bradbury et al., 2007; Chang et al., 2015; Zhang et al., 2016, 2018). A typical initial step in plant GWAS is to order some HapMap accessions’ seeds and characterize their offspring’s phenotypic traits of offspring. However, the considerably high rate of missing and heterozygous genotypes together significantly degrade the specificity of an accession’s original descent. Therefore, before starting an association study, all accessions need to be accurately distinguished and confirmed, especially when the seeds have been propagated for multiple generations, and the heterozygous genetic materials have segregated. The dilemma posed by specificity degradation among accessions make it more difficult for the verification of gene function. Although identifying a specific accession or clear distinguishment of multiple accessions should be the first step in HapMap data analysis, to the best of our knowledge, there are no methodologies or tools available for this purpose.

The sequence variant calling of a HapMap population can result in millions of genetic variant markers such as SNPs or INDELs (insertions or deletions) (Durbin et al., 2010). These genetic variants do not only work as a foundation for investigating the relationship between genotypes and phenotypes, but also as a huge resource to distinguish multiple accessions. In self-pollinated species, a homozygote’s genetic material will be stably conserved in their offspring, and the different homogenous genotypes of a variant marker, being polymorphic, can be used to distinguish one accession set from the others. Several related variant markers can be cascaded, promising to construct a hierarchical decision tree and distinguish a specific accession from the others.

Based on the above presumptions, we propose a novel algorithm, inspired by the Set partitioning in hierarchical tree (SPIHT) algorithm for encoding the discrete wavelet transform (DWT) coefficients in image compressing (Said and Pearlman, 1996). In our algorithm, set partitioning and a hierarchical decision tree are used to distinguish multiple accessions in a HapMap population. Based on the difference in homozygous genotype values, a selected marker can partition an accession set into several distinctive subsets. If multiple genotype variant markers are used, the accession set partitioning will recursively proceed until all accessions are specifically distinguished or until the procedure meets preset criteria. Along with the recursively accession set partitioning, a hierarchical decision tree that records the procedure detail can be constructed.

We implemented the proposed algorithms as a standalone Linux tool using C^++^. Also, we developed a demonstrative web-based tool entitled MAD-HiDTree (Multiple Accession Distinguishment-Hierarchical Decision Tree), which is publicly available at https://bioinfo.noble.org/MAD-HiDTree. MAD-HiDTree will recursively partition the accession set and produce a constructed hierarchical decision tree after receiving the user uploaded matrix data of genotype markers. It will record the specific path of selected markers and homozygous genotypes for one particular subset or accession, distinguishing this subset or accession from the others. We used the *Medicago truncatula* HapMap^1^ data for a case study. To feasibly detect and validate the genetic marker’s polymorphism, we favor INDELs, especially long INDELs, because the presence and absence of an INDEL marker can be easily visualized with PCR (polymerase chain reaction) (Mills et al., 2006). After extracting INDEL markers and filtering out low-quality markers, we generated a genotype marker matrix with 262 accessions and 1,386 markers. Based on the extracted genotype marker matrix in a dimension of 1,386 × 262, MAD-HiDTree can successfully build a hierarchical decision tree and clearly distinguish the original 262 accessions.

## Materials and Methods

### Genotype Marker Matrix Data

The genotype marker matrix in.txt format is the only required input for MAD-HiDTree. The columns of the genotype marker matrix correspond to the individuals/accessions, and the rows correspond to the genetic or genotype variant markers. The matrix entry at a specific row (marker) and column (accession) is the recorded genotype value, which can be either homogenous alleles “0/0,” “1/1,” heterogeneous allele “0/1” or a missing/unknown genotype value “./.”. Here, the allele “0” represents the reference allele, while “1” represents the first alternative allele, which strictly follows the Variant Call Format (vcf) specification^2^.

### HapMap Variant Calling Data

After the initial sequence alignment and genetic variant calling, the HapMap data are usually stored in the sequence alignment/map (SAM) format, or the variant format of either.vcf or its binary version, .bcf (Li et al., 2009; McKenna et al., 2010). The .vcf/.bcf file functionally contains a meta-information header line and the following marker data lines, and can be accessed and manipulated using BCFtools^3^. BCFtools is a set of utilities (Danecek and McCarthy, 2016), through which a.vcf file can be converted to the genotype matrix file. We developed a user-friendly web-based pipeline to facilitate the creation of the genotype marker matrix file by extracting and filtering information from the HapMap variant calling file in a.vcf/.bcf file called GMEF (genotype marker extracting and filtering), based on the BCFtools command lines. The GMEF is publicly available at https://bioinfo.noble.org/GMEF/.

### The Rationale to Distinguish Multiple Accessions Using Homozygous Genotypes

The genotype marker matrix is the only required input data. This can be generated from the original HapMap genetic variant data (in.vcf or.bcf format) by BCFtools or GMEF. As described above, the genotype marker matrix columns correspond to multiple accessions while the rows correspond to the genotype markers. For one accession, the status of a homogenous genotype at one genetic marker can be stably passed onto their descendants, and the distinctive information of two or more homogenous genotype values at the same locus can be used to partition the accession set into different subsets. Multiple markers can be cascaded, promising to construct a hierarchical decision tree and distinguish one specific accession against others.

**FIGURE 1:**
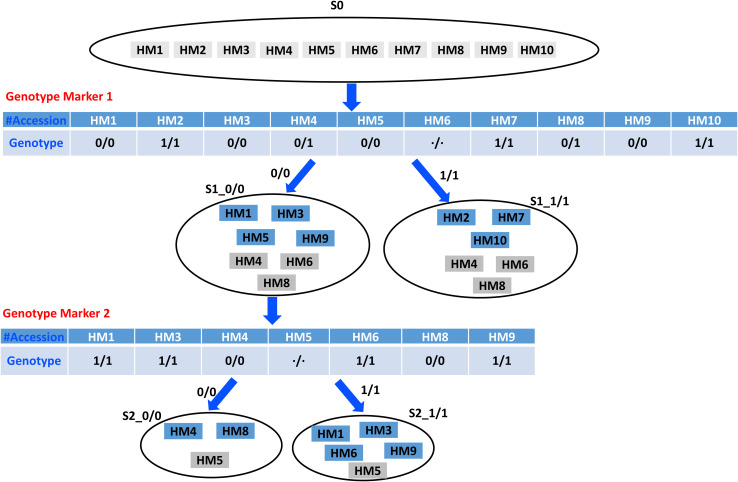
An example on distinguishing 10 HapMap accessions based on the homozygous genotypes of two genotype markers.

Figure 1 depicts the rationale on distinguishing the example 10 HapMap accessions based on the homozygous genotypes of two genotype markers. It essentially uses the homozygous genotypes to partition an accession set and multiple markers to acquire more specific subsets. The original input set of 10 HapMap accessions (HM1–HM10) is partitioned into two subsets by genotype marker 1, and the two partitioned subsets are labeled as S1_0/0 and S1_1/1, containing seven and six accessions, respectively, which, compared with the original input set with 10 accessions, has become more specific. The intermediate subset S1_0/0 is partitioned into two subsets by genotype marker 2, and the two partitioned subsets are labeled as S2_0/0 and S2_1/1, which contain three and five accessions, respectively. We allocate all the accessions with unknown and heterogeneous genotype values into both distinctive subsets, fundamentally allowing a partial overlap for information redundancy. Also, Figure 1 shows that the homozygous genotype marker’s distinctive status is used to partition an accession set. Therefore, an optimal marker requires more even distribution of homozygous genotypes across the accessions and fewer unknown and heterogeneous genotype values.

### Algorithm for Partitioning Accession Set and Constructing Hierarchical Decision Tree

We use the two or more distinctive homozygous genotypes of a selected genotype marker to partition the accession set into two or more distinct subsets. We continuously select more genotype markers and proceed with the above accession set partitioning recursively until a preset threshold or single accession is met, achieving the goal of distinguishing multiple accessions. Meanwhile, as accession set partitioning, we construct a hierarchical decision tree containing all partitioning details.

**FIGURE 2:**
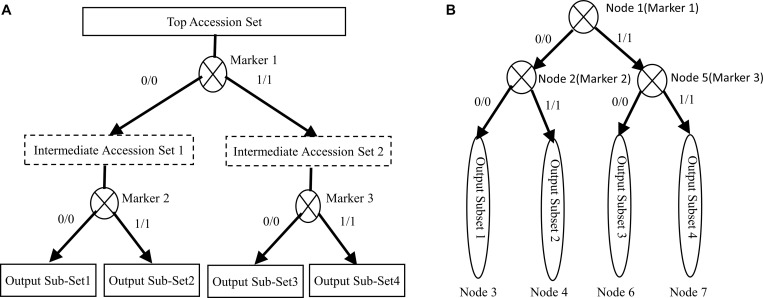
Illustration of the proposed algorithm. **(A)** Partitioning an accession set. **(B)** Construction of a hierarchical decision tree.

Figure 2 illustrates the entire process of the accession set partitioning and the accompanying hierarchical decision tree construction. In Figure 2A, the top accession set is recursively partitioned into four subsets by three markers. Figure 2B shows the corresponding hierarchical decision tree consisting of three marker nodes and four subset nodes. Also, Figure 2 shows that the constructed hierarchical decision tree consists of two types of nodes: the intermediate nodes representing genotype markers and leaf nodes representing the final resulting subsets or specific accessions. We can use the recorded node marker’s homozygous genotype values to design the biological validation experiments and specifically validate and distinguish each accessions.

The principle is to use the least amount of markers to distinguish all the accessions; therefore, the selected marker should have better capability than other markers to distinguish the accession set. Here, we propose a distinguishing score (DS) to measure a selected marker’s distinguishing capability:

(1) $$D\,S=\frac{A\,N-U\,N-H\,N}{A\,N}\,\sum_{i=0}^{T\,G-1}\sum_{j=i\,1}^{T\,G}P_{i/i}^{*}\,P_{j/j}$$

Where A*N*, *UN*, *HN* and *TG* represent the number of all accessions, the number of accessions with unknown genotypes, the number of accessions with heterogeneous genotypes and the number of homozygous genotypes, respectively. The *i*/*i* and *j*/*j* represent the two alleles of each homozygous genotype, while *P*_i/i_, and *P*_j/j_ are the percentage of homozygous genotype *i*/*i* and *j*/*j*, respectively.

For a bi-allelic marker, there will be only two homozygous genotype status: “0/0” and “1/1.” The formula to calculate DS can be simplified as:

(2) $$D\,S=\frac{A\,N-U\,N-H\,N}{A\,N}\,P_{0/0}*P_{1/1}$$

In an ideal case, where *UN* = 0 and *HN=0*, only homozygous genotypes remain, and the percentages of the two homozygous genotypes meet:

(3) $$P_{0/0}+P_{1/1}=1.0$$

It is easy to acquire the maximum DS score for a bi-allelic genotype marker as:

$$D\,S=0.25,\text{when}\,P_{0/0}=P_{1/1}=0.5.$$

From the formulas 1–3, we see that the above mathematical definition of DS can fairly measure the marker’s distinguishing capability. For one marker, the fewer unknown and heterogeneous genotypes and the more even the distribution of homogenous genotypes across all the accessions is, the higher the DS score.

**FIGURE 3:**
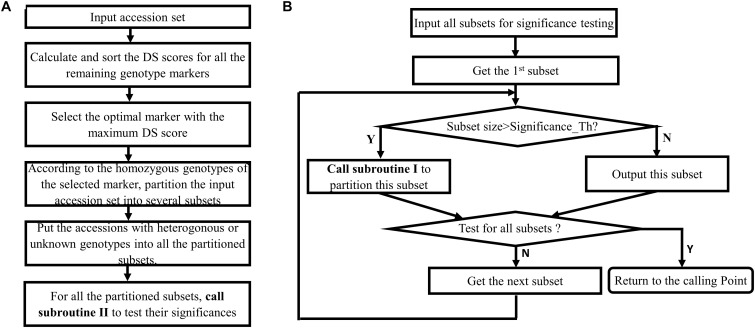
Flowchart of the proposed algorithm that includes two cross-related subroutines. **(A)** Subroutine I for partitioning an accession set. **(B)** Subroutine II for testing the significance of accession subset.

Figure 1 provides the rationale for using different homozygous genotype status of a genotype marker to partition a larger accession set into more-specific smaller accession subsets. Figure 2 shows that the multiple selected markers can be used to partition the top accession set to a discernable level recursively, and the information recorded in the accompanied hierarchical decision tree can be used to design the biological experiment for verification. The algorithm for accession set partitioning and hierarchical decision tree construction can be refined and dissected into two separate but cross-related subroutines: subroutine I for accession set partitioning and subroutine II for subset significance testing. Figure 3 depicts the flowchart between the two subroutines.

Subroutine I is for partitioning an accession set, applying to all of accession sets to be partitioned and can be described as following:

1. Calculate and sort the DS scores for all the genotype markers.
2. Select the optimal marker with the maximum DS score.
3. Partition the inputting accession set into different subsets according to the distinctive homozygous genotypes.
4. Allocate the accessions with heterogonous or unknown genotypes into one of the partitioned subsets.
5. Call subroutine II (Figure 3B) to test the significance of the partitioned subsets.

The subroutine II is called by subroutine I to test the significances of the partitioned subsets, and can be described as following:

1. Test the significance of the first subset:
  If the current subset size is larger than the preset threshold, recursively call subroutine I to partition the current subset. Otherwise, output this subset.
2. If any subsets that have been not tested, get the next subset for testing. Otherwise, return to the calling point.

Essentially, the whole procedure is an iterative and recursive process for accession set partitioning and subset significance testing. A hierarchical decision tree will be gradually constructed during the accession set partitioning process. In the SPIHT algorithm for image compressing, the DWT coefficients are recursively partitioned into subsets, and the topology architecture is organized as a hierarchical tree. A subset’s significance is determined by the maximum of the absolute values of the wavelet coefficients in the subset (Said and Pearlman, 1996). Inspired by SPIHT, we modified and utilized this concept in HapMap data analysis to distinguish multiple accessions. Here, the significance of a subset is simply defined as the accession number.

Once the partitioning the accession set is finished, the hierarchical decision tree consisting of the selected genotype markers as intermediate nodes, homozygous genotype values labeled in branches, and the final output accessions or subsets as terminal nodes, will be output. If we set the significance threshold as 1, we can partition the accession set completely and output the specific distinguished individual accessions.

The above description indicates that the accession set partitioning by bi-allelic genotype markers will result in a full binary tree in which every node in the tree has either zero or two children (Black, 2014). Therefore, a perfect scenario to partition 2^*M*^ (*M* is an integer) accessions by perfect bi-allelic genotype markers will produce a perfect binary tree. Here, the perfect bi-allelic genotype marker means that it can partition the accession set into two equal subsets, and there are absolutely no accessions labeled as unknown or heterogonous genotype values. The perfect binary tree means that all intermediate marker nodes have two children, while all leaf nodes have only one specific accession and the same depth (Black, 2019). In this ideal scenario, 2^*M*^−1 perfect bi-allelic markers will completely partition the original 2^*M*^ accessions. If we preset the subset significance as a size of 2^*N*^ (*N* is another integer), the set partitioning of original 2^*M*^accessions will need 2^M−N^−1 perfect bi-allelic markers, and the final constructed hierarchical decision tree will consist of 2^M−N^−1 intermediate marker nodes and 2^M−N^ terminal subset nodes.

### Design and Implementation

**FIGURE 4:**
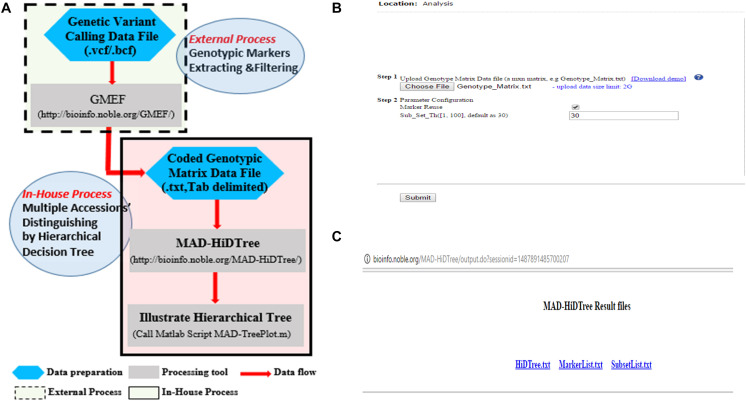
Design and implementation of MAD-HiDTree. **(A)** Flowchart of data analysis using MAD-HiDTree at different scenarios. **(B)** The MAD-HiDTree web user-interface for data submission and parameter configurations. **(C)** The output page of MAD-HiDTree.

To distinguish multiple accessions in a HapMap population, we used the distinctive homozygous genotypes of genetic markers to partition the top accession set and construct a hierarchical decision tree. To tolerate the considerable amount of missing and heterogeneous genotypes, we adopted a strategy to allocate them into the distinctive subsets. The genotype marker matrix data is the only input data, which can be acquired from the original genetic variant calling HapMap file by the command lines of BCFtools. We developed MAD-HiDTree to distinguish multiple accessions. We also developed several peripheral tools and related scripts to assist the analysis in different scenarios. Figure 4A depicts an overview of how to use MAD-HiDTree. In scenario 1, the genotype matrix data is available; therefore, the user only needs to submit the genotype marker matrix data to MAD-HiDTree and call a Matlab script to illustrate the generated hierarchical decision tree. In scenario 2, the user can use the original HapMap genetic variant calling data in either.bcf or.vcf format to create the genotype marker matrix data through command lines of BCFtools or our GMEF web tool. We developed GMEF based on the command lines of BCFtools. GMEF also hosts several versions of *M. truncatula* HapMap data, and we are committed to hosting other HapMap data upon user requests.

We rationally designed the MAD-HiDTree into three function modules: accession set partitioning, accession subset significance testing, and DS score calculation. The first two function modules are recursively called by each other, so inherent complexity does exist. We chose standard C/C^++^ as the programming language and used Standard Template Libraries (STL) for implementation. All codes were written and compiled using Code::Blocks in a Linux environment.

MAD-HiDTree only requires three inputting parameters: one text file for the genotype marker matrix, one bool variable to indicate whether reusing the selected marker and one integer variable to specify the output subset significance size. When one genotype marker is chosen for accession set partitioning, the marker and the homogenous genotypes are recorded in the constructed hierarchical decision tree. Logically, a used marker can be reused in the later subset partitioning, but the user can opt not to reuse the marker. The preset subset significance threshold determines the outputting subset size and the recursion depth. Suppose we set the output subset significance threshold as one and the available markers are large enough. In that case, MAD-HiDTree will recursively partition the accession set and finally output the specific distinguished accessions. As we know, the recursion depth determines the stack level and also affects memory consumption.

When the analysis is completed, MAD-HiDTree will generate three text files: one file to record the generated hierarchical decision tree, and the other two files to record the used marker index list and accession list for each output subset, respectively. We also developed two Matlab scripts to visualize the generated hierarchical decision tree. One script is responsible for reading and converting the information from the.txt file. The other script is responsible for displaying the tree and annotating the homozygous genotypes, using the Matlab “tree layout” function.

## Results

### Submission of Representative Genotype Maker Matrix Data to MAD-HiDTree

An example INDEL genotype marker matrix (Supplementary File 1) was extracted from our hosted *M. truncatula* HapMap data and stored as a genotype marker matrix in a dimension of *A* = 1386 × 262. This data was further used to demonstrate and evaluate the performance of MAD-HiDTree in different scenarios. On the web page, MAD-HiDTree (Figure 4B) requires the user to configure two simple parameters: whether to reuse markers, and the threshold for outputting subset size. Here, we set the subset size as 30 and choose not to reuse markers. On clicking the “submit” button, the MAD-HiDTree will iteratively and recursively analyze the uploaded data to partition the accession set and construct the hierarchical decision tree. Once the analysis is complete, MAD-HiDTree will display a pop-up window (Figure 4C), prompting the user to download three results files (Supplementary File 2): HiDTree.txt, MarkerList.txt, and SubsetList.txt, which record the information of the generated hierarchical decision tree, the selected marker index list and the final partitioned accession list for each output subset, respectively.

### Illustration and Biological Explanation of MAD-HiDTree Results

**FIGURE 5:**
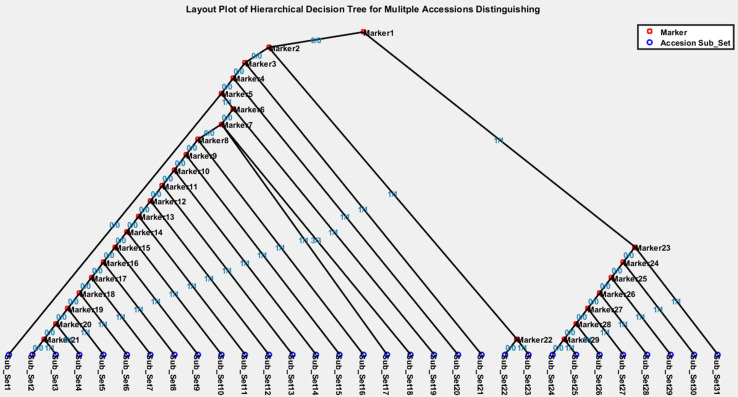
Visualization of an example hierarchical decision tree.

A hierarchical decision tree can be visualized (Figure 5) by our custom Matlab script (Supplementary File 3) that interprets the hierarchical decision tree file, such as “HiDTree.txt,” which can be downloaded from the MAD-HiDTree result output page. Figure 5 illustrates an example hierarchical tree contains 29 intermediate marker nodes and 31 leaf subset nodes. Two different symbols denote the marker nodes and subset nodes. The homozygous genotypes are also labeled as different decision directions. The path from the top node to the subset leaf node demonstrates what genotype markers and homozygous genotypes are needed to distinguish the specific subset from others clearly. For example, subset 1 can be specifically distinguished by “marker 1” under genotype “0/0,” “marker 2” under genotype “0/0,” “marker 3” under genotype “0/0,” “marker 4” under genotype “0/0,” and “marker 5” under genotype “0/0.” In contrast, subset 23 can be specifically distinguished by “marker 1” under genotype “0/0,” “marker 2” under genotype “1/1,” and “marker 22” under genotype “1/1.”

The “SubsetList.txt” output file records all the output subsets, along with the information of the contained accessions and the path information from the top node to the leaf node for each subset. The selected markers for the hierarchical decision tree can be retrieved and mapped to the original row/index in the genotype marker matrix through the “MarkerList.txt” output file.

In short, the three returned files from MAD-HiDTree provide the comprehensive information needed for the distinguishing of the multiple accessions, and the layout figure by the Matlab script offers an intuitive and straightforward decision tree view that visualizes the distinguishment of multiple accessions.

### Performance Evaluation

We developed the MAD-HiDTree specifically to distinguish multiple accessions in a HapMap population. In principle, it only requires a set of genotype marker matrix data and two simple parameters to be configured. The two parameters include a bool variable to indicate whether reusing the selected markers and an integer variable to define the output subset size. When setting the integer variable for the subset size as 1, individual accession will be precisely determined. We used the same genotype marker matrix data with 1,386 markers and 262 accessions and submitted it to MAD-HiDTree in different combinations of the two parameters. We recorded all MAD-HiDTree returned results, the two types of the hierarchical decision tree’s node components as markers and subsets, and the entire running time at different parameter combinations. Table 1 shows these performance evaluation results.

**TABLE 1:** MAD-HiDTree performance evaluation at different parameter settings.

Parameters		Performance		
	
Marker reuse	Subset size	No. markers	No. subsets	Running time (s)
No	30	29	31	4
Yes	30	26	29	3
No	20	42	44	5
Yes	20	37	40	4
No	10	73	75	7
Yes	10	65	68	6
No	5	124	126	11
Yes	5	120	123	10
No	1	351	347	22
Yes	1	359	362	25

Table 1 shows that the preset subset size threshold greatly affects the number of markers, the number of outputting subsets, and the running time. The smaller the subset size, the more markers used, and the more specific output subsets and the longer the running time will be. The parameter option of whether to reuse a marker in the subsequent accession set partitioning only moderately affects the overall performance. Because the accessions with unknown genotypes or heterogeneous genotypes are put into all the partitioned subsets with distinctive homozygous genotypes, information redundancy does exist, and some accessions may be classified into multiple output subsets. When we preset the output subset size as 1, we find that it is not the original 262, but rather 347 and 362 specific accessions that are output in the two scenarios: to reuse and not reuse the marker, respectively. In application, one can choose any one of the decision paths for the repeated accessions; the recorded markers and homozygous genotypes can be used to identify one accession from other accessions.

### Biological Experiment Validation

To biologically validate the proposed method and that the extracted markers can be used to distinguish a specific accession, we need to set the subset size as 1, which will let MAD-HiDTree generate a very deep hierarchical decision tree. In this scenario, each leaf node represents a specific accession and has a corresponding path starting from the root node. We can specifically identify an accession if we validate all the markers and homozygous genotypes of a specific path.

The original Medicago Hapmap population contains 262 accessions. The accession HM101 (A17) was used as the reference for genome sequencing. Therefore, all the homozygous alleles from HM101 are labeled as “REF,” otherwise labeled as “ALT” indicating the polymorphism. We selected accession HM014 as an experiment example and searched all the marker-homozygous genotype paths from the very beginning root node to the leaf node representing accession HM014. The marker-genotype path containing 9 related INDELs that determine the accession HM014. We extracted the 9 related INDEL markers and recorded the corresponding information (Supplementary Table 1). The recorded homozygous genotypes (Supplementary Table 2), “0/0” and “1/1” means the alleles between accessions HM014 and HM101 are exactly the same or different, respectively, need to be validated one by one. Based on this INDEL information, we accessed the flanking sequences around the 9 INDEL markers and designed the forward and reverse primers (Supplementary Table 3). The uniqueness of the primer’s sequence in whole genome should be carefully checked because it essentially determines that the PCR product covering the sequence gap in the INDEL marker can be successfully amplified.

We extracted the gDNA from the leaf tissue, and each accession had three plants. The description of Medicago Hapmap accessions and the method to sample and extract gDNA can be referenced in our previous publication (Supplementary Document 1). Following the manufacturer’s protocol, we used Ex Taq (Takara Bio Inc.) PCR amplification. The resulting PCR products of the 9 selected INDEL markers are illustrated in Supplementary Figure 1. From Supplementary Figure 1, we can verify the correctness of the marker-homozygous genotype chain: (1,007; 1/1)→(623; 1/1)→(90; 0/0)→(897; 0/0)→(798; 1/1)→(913; 0/0)→(727; 1/1)→(422; 1/1)→(284 0/0). Obviously, there is no difference between HM014 and A17 at markers #90, #897, #913, and #284, and discernable difference at marker #1,007, #623, #798, #727, and #422. The results indicate that we can use the two typical classes of homozygous genotype as 0/0 and 1/1, for the partition of the accession subset. For an unknown Medicago Hapmap accession, if we verify the above marker-homozygous genotype path and get similar PCR results as in Supplementary Figure 1, we can confidently claim it as accession HM014. In real applications, we can extend the above method and procedure to identify other accessions.

Further, once we verify all of the possible marker-homozygous genotype paths of the hierarchical decision tree, we can claim that we have identified the entire Hapmap accessions. We are aiming to achieve this goal and realize the identification of the whole Medicago Hapmap accessions in the future. At the same time, we will collect the phenotypes for each HapMap accession, such as pictures of each accession at different growing periods, different tissues, etc. We envision that the combination of molecular markers and phenotype information will provide more comprehensive information for HapMap accession distinguishing, identification, and validation.

## Discussion

When design the algorithm to distinguish multiple accessions, we presumed the considerately high accuracy of the final genotype marker matrix, and the stable conservation of homozygote’s genetic material among their offspring. Regarding the case study using the Medicago Hapmap data to identify *Medicago* accessions, we chose not SNP but INDEL to generate the genotype marker matrix, because we think that it could be easy to validate INDEL markers’ polymorphisms based on the PCR product size.

In real application, some recorded homozygous genotype number may be wrong due to base-calling and/or alignment errors because of the limitations in sequencing technology when generating the Medicago HapMap data over a decade ago. Therefore, we need to check the correctness of each marker’s genotype number among multiple accessions. Once confirmed, we need to remove and/or replace the markers with wrongly called genotype number from the genotype matrix.

Once the hierarchical decision tree is constructed, the PCR experiment for validation will proceed. The difference of the INDEL’s polymorphism reflecting PCR product size should be carefully considered, which is indeed determined by the molecular experiment detection capability and wet-lab researchers’ experience. Additionally, researchers need to consider the specificity of the genome region when designing the primer sequences. Furthermore, considering the nature of the established decision tree’s hierarchical structure, we suggest testing and validating the decision path from the root of the decision tree, gradually adjusting and refining some unfit markers, and eventually optimizing all chosen markers.

The genotype marker matrix data that MAD-HiDTree requires can be from any genetic variant markers as long as biological experiments can validate it. It is reported that some practical approaches have been developed to validate both the INDEL and SNP genotype markers’ polymorphism (Kim et al., 2016). In principle, the discernible genotype numerical tags as “0/0,” and “1/1” are only used to classify and partition the accessions into different accession groups. Therefore, if we can avoid the genetic segregation issues at their offspring in self-pollinated species, we can use heterozygous genotypes to build additional discernible accession groups. For a bi-allelic marker, we only need to use three numerical tags as “0/0,” “1/1,” and “2/2” to represent the two homozygous genotypes and one heterozygous genotype, respectively. Such type of genotype marker matrix can be well supported by our web tool MAD-HiDTree, the analysis result will generate a hierarchical decision tree, which will not a binary tree but a polytree.

## Conclusion

We introduced a novel distinguishing score (DS) to measure a selected marker’s differentiating capability, using homogenous genotypes of a genetic marker to distinguish one accession set against another. Inspired by the set-partitioning theory in computer science, we proposed a new algorithm to distinguish multiple HapMap accessions by recursively partitioning accession sets and constructing a hierarchical decision tree. We successfully developed a demonstrative online tool called MAD-HiDTree, which analyzes the inputting genotype matrix data and construct a hierarchical decision tree by recursively partitioning the accession set. It is worth note that when the integer variable for the subset size is set as 1, individual accession can be precisely determined. The specific path for an output accession or subset is recorded by the selected markers and homozygous genotypes, which can be used to distinguish this accession or subset from others. Using the genotype matrix data extracted from the *M. truncatula* HapMap project, we constructed the hierarchical decision trees at different parameter combinations, efficiently distinguishing the original 262 accessions. We validated one accession with PCR experiments and verified that our proposed method and MAD-HiDTree could identify a specific accession. We can naturally extend the method and procedure to other accessions. Therefore, our method and MAD-HiDTree tool are useful in generating a molecular marker database for distinguishing accessions for HapMap projects.

## Data Availability Statement

The original contributions presented in the study are included in the article/Supplementary Material, further inquiries can be directed to the corresponding author.

## Author Contributions

WZ implemented the accession set partitioning algorithm, developed the web tool MAD-HiDTree, carried out case study analyses and wrote the manuscript. NK collected HapMap data. WZ, YK, W-RS, and PZ conceived of and directed the study. HZ and IT-J provided the sample and materials. XC and JW designed the primer and conducted the PCR experiment validation. WZ, YK, and PZ performed bioinformatics analyses and co-wrote the manuscript. All authors contributed to the article and approved the submitted version.

## Conflict of Interest

The authors declare that the research was conducted in the absence of any commercial or financial relationships that could be construed as a potential conflict of interest.

## Footnotes

1 http://www.medicagohapmap.org/

2 https://github.com/samtools/hts-specs

3 http://github.com/samtools/bcftools

## Supplementary Material

Supplementary File 1 The genotype marker matrix data used to submit to MAD-HiDTree in this study. 1,386 INDEL markers and 262 accessions are contained in this data, originally from the *Medicago truncatula* HapMap variant data.

Supplementary File 2 The MAD-HiDTree analysis result files for the testing genotype marker matrix data. Three.txt files are included, “HiDTree.txt,” “MarkerList.txt” and “SubsetList.txt,” which correspond to the information for hierarchical decision tree, selected marker list, and the subset list, respectively.

Supplementary File 3 The Matlab script packages to plot the layout of the generated hierarchical decision tree. Three files are included: the example file “HiDTree.txt” and two Matlab script files.

## Abbreviations

HapMap: Haplotype Map
G2P: Genotype to Phenotype
SNP: Single Nucleotide Polymorphism
INDEL: Insertion or Deletion
GWASs: Genome-Wide Association Studies
SPIHT: Set Partitioning in Hierarchical Tree
DWT: Discrete Wavelet Transform
PCR: Polymerase Chain Reaction
SAM: Sequence Alignment/Map
VCF: Variant Call Format
BCF: Binary counterpart of VCF
GMEF: Genotype Marker Extracting and Filtering
MAD-HiDTree: Multiple Accession Distinguishment- Hierarchical Decision Tree
DS: Distinguishing Score
STLs: Standard Template Libraries.
