# Supplementary figures and images for: Distinguishing HapMap Accessions Through Recursive Set Partitioning in Hierarchical Decision Trees
Source: Front Plant Sci. 2021 Feb 3;12:628421. doi: 10.3389/fpls.2021.628421 (PMC7886675; doi:10.3389/fpls.2021.628421)

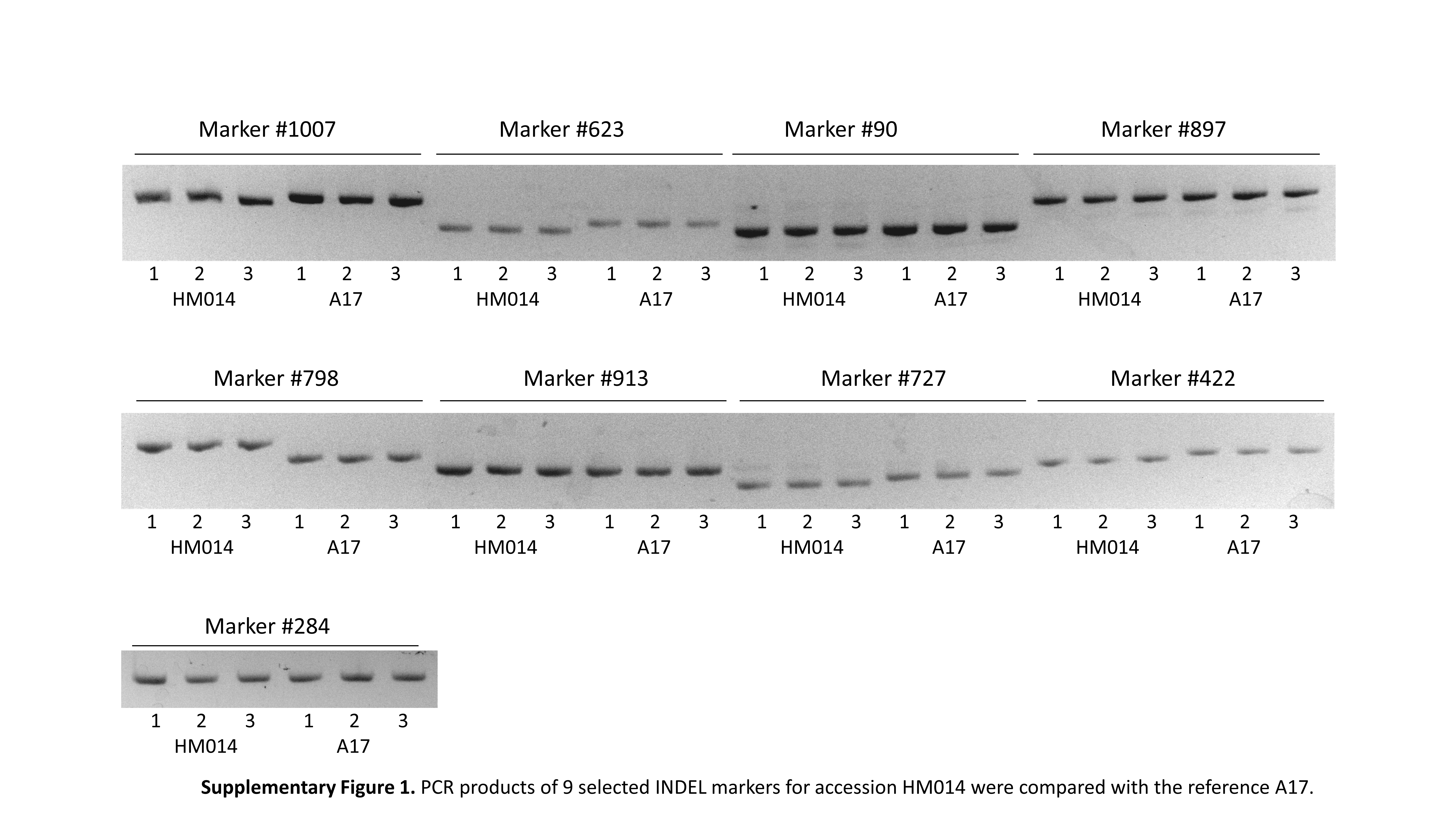

Supplement: Supplementary file 4 [file Image_1.tif]
