# Supplementary material for: Distinguishing HapMap Accessions Through Recursive Set Partitioning in Hierarchical Decision Trees
Source: Front Plant Sci. 2021 Feb 3;12:628421. doi: 10.3389/fpls.2021.628421 (PMC7886675; doi:10.3389/fpls.2021.628421)
Supplement: Supplementary file 5 [file Table_1.pdf]

Supplementary Table 1. Information of 9 INDEL markers that were used to identify accession HM014

# Marker Number	CHROM	POS	REF	ALT
1007	chr8	22801048	TTTTTTGGATTTCAGACAAGCTC	T
623	chr5	3179952	TATCCAAACATACACTG	T
90	chr1	28080088	CTTGACCAT	C
897	chr7	35760753	CCCCCATCCAGTAGGT	C
798	chr7	631963	GCCCATTCCCATA	G
913	chr7	41776856	GCAGTCACAGTCCCATA	G
727	chr5	42721868	ATTGAACATGC	A
422	chr3	50663975	AGTGTAAATCTCC	A
284	chr2	44029260	AAACAGGTTC	A
