# Supplementary material for: Distinguishing HapMap Accessions Through Recursive Set Partitioning in Hierarchical Decision Trees
Source: Front Plant Sci. 2021 Feb 3;12:628421. doi: 10.3389/fpls.2021.628421 (PMC7886675; doi:10.3389/fpls.2021.628421)
Supplement: Supplementary file 6 [file Table_2.pdf]

Supplementary Table 2. Homozygous genotypes of 9 INDEL markers to determine accession HM014

# Marker Number	Homozygous Genotype
1007	1/1
623	1/1
90	0/0
897	0/0
798	1/1
913	0/0
727	1/1
422	1/1
284	0/0
