# Supplementary material for: Distinguishing HapMap Accessions Through Recursive Set Partitioning in Hierarchical Decision Trees
Source: Front Plant Sci. 2021 Feb 3;12:628421. doi: 10.3389/fpls.2021.628421 (PMC7886675; doi:10.3389/fpls.2021.628421)
Supplement: Supplementary file 7 [file Table_3.pdf]

Supplementary Table 3. Sequences of primers used for PCR amplification to identify accession HM014

# Marker Number	Genotype	Forward Primer (5’ to 3’)	Reverse Primer (5’ to 3’)
1007	1/1	CACGGGGGCACAAACTCCT	TGCAGTGGAATGCTAGGGC
623	1/1	TCCTTTGAACGTTTAGAGTTG	TTGGCATGCAGGTGTGGA
90	0/0	GTCTAGCTCAGCTTGACCA	CATGAACAATCAAGCCGAT
897	0/0	CACGATACTGTTCCCAAGAG	TCAGGATGTGGAAAGAGAAG
798	1/1	TGGTGGAAACTAGGCTTAGGT	CAAGCAATGTCTGTGTCACTG
913	0/0	CTGGAGGTGGATTCATTCT	CAATTGGTTAAGATCGAACAGT
727	1/1	AGCCTCTGAGGTGTCAAGT	TATCCTAAAGGTCTAGCCAC
422	1/1	CAACTCAACTGGTGCCTC	CATTGATGGCAGAACCAAC
284	0/0	GATGTTTGGCAGTGGTTG	GTATCAATAACACCACGCAG
