# Supplementary material for: Distinguishing HapMap Accessions Through Recursive Set Partitioning in Hierarchical Decision Trees
Source: Front Plant Sci. 2021 Feb 3;12:628421. doi: 10.3389/fpls.2021.628421 (PMC7886675; doi:10.3389/fpls.2021.628421)
Supplement: Supplementary file 8 [file Presentation_1.pdf]

Distinguishing HapMap Accessions through Recursive Set Partitioning in Hierarchical Decision Tree

Wenchao Zhang, Yun Kang, Xiaofei Cheng, Jiangqi Wen, Hongying Zhang, Ivone Torres-Jerez, Nick Krom, Michael K Udvardi, Wolf-Rüdiger Scheible and Patrick Xuechun Zhao*

Noble Research Institute LLC, 2510 Sam Noble Parkway, Ardmore, OK 73401, USA.

***Author of correspondence**

Patrick X. Zhao, pzhao@noble.org Phone: +1-580-224-6725

Keywords: Genome-wide association study, genotype, HapMap accession, homozygous, hierarchical decision tree, INDEL, set partitioning, SNP.

Supplementary Document

Description of Medicago HapMap Accessions

The HapMap population was sequenced by Illumina sequencing using genomic DNAs pooled from 30-50 plants each line. Before sequencing, each line had been selfed for a minimum of three generations. The sequencing depth was 20X for the 30 deeply sequenced lines and at least 5X for the rest lines.

In the *M. truncatula* HapMap population, the parent plants used in genomic sequencing were selfed for a relatively low number of generations and multiple plants were pooled together for genomic DNA extraction, therefore, it can be observe a high level of heterozygosity in the HapMap SNPs or INDELs.

NGS short reads were aligned to the *M. truncatula* v4.0 reference genome, representing the A17 genotype (HM101, Young et al, 2011). Alignment and SNP/INDEL calling was performed at NCGR using GSNAP (Wu et al, 2010) and GATK (McKenna, Aaron, et al, 2010). GSNAP (version 20130331) was used to produce initial bam alignment files, and the standard GATK pipeline was used to process the aligned data to generate the genetic variants. The resulting variant calls were marked in a VCF file.

gDNA extraction and PCR Experiment for HM014 and A17

Leaves from three individual plants of HM014 and A17 were sampled as three biological replicates and genomic DNA was extracted according to the method described previously (Cheng et al. 2011). ExTaq (Takara Bio Inc.) was used for PCR amplification following the manufacturer's protocol. PCR cycles are: 95°C 30 sec; 94°C 15 sec, 58°C 30 sec, 72°C 1 min, for 5 cycles; 94°C 15 sec, 56°C 30 sec, 72°C 1 min, for 5 cycles; 94°C 15 sec, 54°C 30 sec, 72°C 1 min, for 25 cycles; 72°C 5 min, stored at 10 °C. 10 μl of PCR reaction was electrophoresis on 3.5% agarose gel.

References

[1] Young, Nevin D., et al. "The Medicago genome provides insight into the evolution of rhizobial symbioses." *Nature* 480.7378 (2011): 520-524.

[2] Wu, Thomas D., and Serban Nacu. "Fast and SNP-tolerant detection of complex variants and splicing in short reads." *Bioinformatics* 26.7 (2010): 873-881.

[3] McKenna, Aaron, et al. "The Genome Analysis Toolkit: a MapReduce framework for analyzing next-generation DNA sequencing data." *Genome research* 20.9 (2010): 1297-1303.

[4] Cheng X, Wen J, Tadege M, Ratet P and Mysore KS. Reverse genetics in *Medicago truncatula* using Tnt1 insertion mutants. Methods in Molecular Biology, 2011, **678**: 179-190.
